# Feasibility of Computational Fluid Dynamics for Evaluating the Intraventricular Hemodynamics in Single Right Ventricle Based on Echocardiographic Images

**DOI:** 10.1155/2018/1042038

**Published:** 2018-01-16

**Authors:** Li-Jun Chen, Zhi-Rong Tong, Qian Wang, Yu-Qi Zhang, Jin-Long Liu

**Affiliations:** ^1^Department of Pediatric Cardiology, Shanghai Children's Medical Center, Shanghai Jiaotong University School of Medicine, Shanghai 200127, China; ^2^Department of Cardiothoracic Surgery, Shanghai Children's Medical Center, Shanghai Jiaotong University School of Medicine, Shanghai 200127, China; ^3^Institute of Pediatric Translational Medicine, Shanghai Children's Medical Center, Shanghai Jiaotong University School of Medicine, Shanghai 200127, China; ^4^Department of Medical Imaging, Shanghai Children's Medical Center, Shanghai Jiaotong University School of Medicine, Shanghai 200127, China

## Abstract

This study introduced a combined computational fluid dynamics (CFD) and echocardiography methodology to simulate blood flow in the single right ventricle (SRV) and normal ventricles to study the intraventricular flow. Derived from echocardiographic image loops, CFD-based three-dimensional (3D) flow models of normal subject's left ventricle (LV) and right ventricle (RV) and SRV with and without heart failure at three characteristic diastolic statuses were reconstructed. The CFD derived morphological and functional measurements in normal ventricles and the SRV were validated with echocardiography. The vortex in the normal ventricles and the SRV were studied. The morphological and functional measurements derived from CFD modeling and echocardiography were comparable, and both methods demonstrated the larger volume and higher spherical index in the SRV, in particular the SRV with heart failure. All the vortices in the SRV were smaller than those in the normal control subject's LV and RV, notably with heart failure. Unlike normal LV and RV, no vortex ring was observed in the SRV. Echocardiography-based CFD demonstrated the feasibility of quantifying ventricular morphology and function; in addition, CFD can detect the abnormal flow pattern (smaller or obliterated vortices) in the SRV when compared with normal ventricles.

## 1. Introduction

Single right ventricle (SRV) is a congenital heart disease which causes severe cyanosis. SRV exhibits a large right ventricle as the dominant ventricular chamber (DVC) and a hypoplastic left ventricle as the rudimentary chamber (RC). The SRV has the right ventricle anatomy but works as both pulmonic and systemic circulation ventricles; it is usually unable to sustain separate pulmonary and systemic circulations in sequence, nevertheless offering normal ventricular performance. The SRV has impaired systolic and diastolic function compared with that of the respective ventricles in a normal biventricular heart [1].

Systolic dysfunction of the ventricle accompanied with abnormal diastolic function can be gauged by the ventricular filling impairment [2]. The Working Group for the European Society of Cardiology proposed that diagnosis of diastolic heart failure requires not only presence of clinical signs or symptoms, but also evidence of abnormal filling [3]. Visualization of the flow pattern inside the ventricle during filling phase provides incremental value of understanding the flow dynamics; it also allows better insights into both physiologic and pathophysiologic process during the cardiac cycle. Analysis of the properties of ventricular flow can prompt early detection of cardiac dysfunctions [4, 5].

Four-dimensional flow cardiac magnetic resonance (CMR) can visualize intracardiac flow characteristics in the heart and vessels [6], but the poor temporal resolution of CMR only allows flow visualization based on the averaged flow signals over several cardiac cycles [6], which cannot provide real-time dynamic flow information. Vector flow mapping (VFM) and Echo particle imaging velocimetry (EPIV) both are based on echocardiography (Echo) images and allow visualization of flow in the left ventricle [7, 8], but the inherently low temporal and spatial resolution from Echo limit their uses. Cardiac catheterization and radionuclide angiography were not routinely used to visualize the cardiac flow due to radiation.

Computational fluid dynamics (CFD) can investigate blood flow dynamics in the vessel as well as in the ventricle and atrium [9, 10]. CFD can analyze blood patterns in any heart with any anatomic abnormalities due to no limitations of geometry assumption. To our knowledge, this is the first study on intraventricular flow of single right ventricle using CFD.

This study aimed to provide a primary step in the numerical simulation of fluid dynamics in a SRV with and without heart failure and to compare them with those of normal left and right ventricle using CFD, in particular, to analyze vortices during diastole to provide detailed information for physicians to give patients timely intervention as soon as possible.

## 2. Methods

The study was performed on a male subject who was diagnosed with SRV at two stages (with and without heart failure), and an age- and gender-matched normal child (Table 1). The parents of the study children gave informed consent. The study design, manner of data collection, and analysis were approved by the local institutional review board (IRB) and regional research ethics committee (REC) of Shanghai Children's Medical Center Affiliated to Shanghai Jiaotong University School of Medicine. All the methods in this study were performed in accordance with the IRB and REC guidelines and regulations.

**Figure 1:**
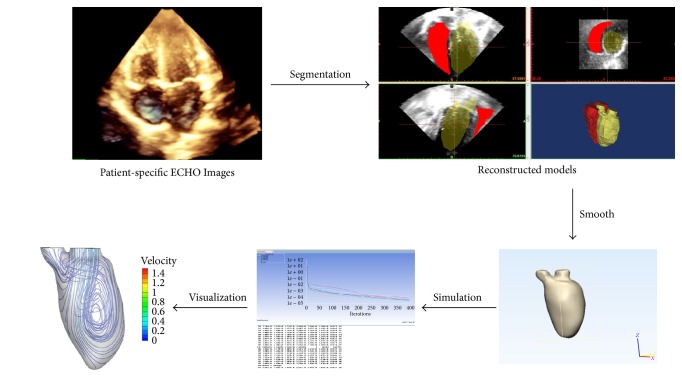
*The process of CFD 3D model reconstruction and simulation*. The CFD modeling is based on real-time 3D echocardiography. A smith dynamic geometry was used to reconstruct the 3D ventricular geometry. The reconstructed model was smoothed by the “Smooth Mask” tool. The reconstructed ventricular geometry along with the boundary conditions were imported into the software (ANSYS®-FLUENT) for simulation. Finally, the visualization of the streamlines was completed by the ANSYS CFD-Post 14.5 software.

Full-volume, three-dimensional (3D) echocardiographic image loops of the heart were obtained by an IE33 ultrasound machine (Philips, Andover, MA, USA) equipped with a 3D transducer (X5-1). The transducer frequency (1.6–3.2 MHz) was optimized for image acquisition. The image depth (10 cm) and sector width (110°) were adjusted to maximize the frame rate (>30 frames/second). The echo image loops were used to reconstruct the chamber geometry and then for further simulations. Echo image segmentation was achieved by using medical imaging software (Materialise®-Mimics 17.5, Plymouth, MI, USA) and then the 3D ventricular model was reconstructed (Figure 1). Initially, the full-volume echo images were imported into the software; then a “Thresholding” tool was applied to specify the range of the ventricular wall. After defining the ventricular wall, a “Multiple Slice Edit” tool was applied to edit multiplane images to remove the surrounding noncardiac structures. An addition “Edit Masks” tool will be employed if the automatic identification for the cardiac anatomy was unclear. The “Region Growing” tool was then used to generate the 3D ventricular model; once the model was established the “Smooth Mask” tool was used to smooth the 3D model for subsequent simulations. The papillary muscles and valves for the 3D model were smoothed out.

**Figure 2:**
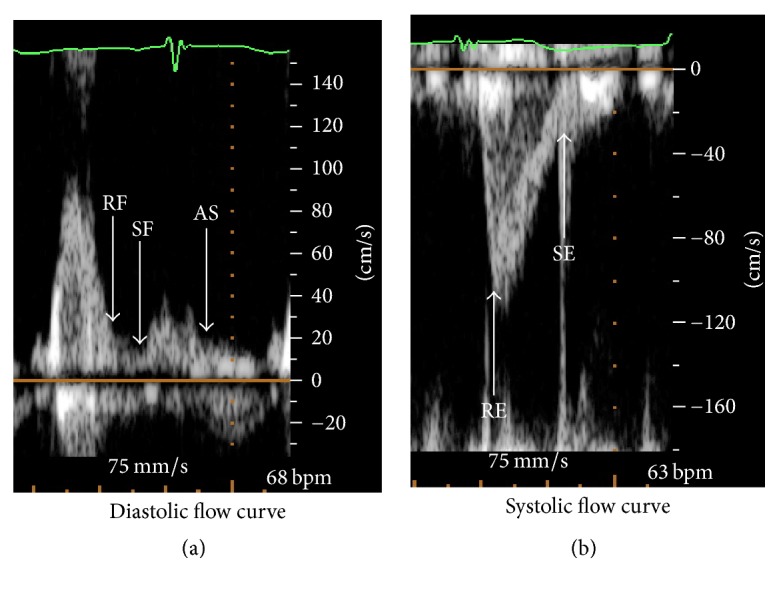
*Boundary conditions of the CFD-simulation models from echocardiographic flow curve*. The inflow/outflow boundary conditions used for simulation were obtained from the mitral/aortic valve velocities by echocardiography at the end of five characteristic periods. (RF: rapid filling; SF: slow filling; AS: atrial systole; RE: rapid ejection; SE: slow ejection. Arrows are at the end of five periods).

In addition to the full-volume 3D echo image loops, 2D echo image loops were acquired with a S5-1 transducer at >60 frames per second. The pulse wave Doppler was used to determine the flow curve at the inlet and outlet of LV (Figure 2, Table 2). The velocities of the inlet and the outlets of the LV were imported into the software (ANSYS-FLUENT 17.0) as the boundary conditions for CFD simulations.

The cardiac anatomy was determined by echocardiography and then represented by five body-fitted prism layers as boundaries (ANSYS-ICEM 17.0) for numerical solutions of the Navier–Stokes flow equations. The distance from the first layers to the model surface was set as 0.02 mm. A tetrahedral mesh, containing approximately 1,000,000 to 3,000,000 tetrahedral mesh elements, was used to fill the remainder of the calculated domain. The computational domain was extended to include the proximal atrium and ascending aorta or pulmonary artery for accurate simulation and calculation. The simulation was accomplished by using the ventricular geometry for reconstruction and the velocities of inflow/outflow as boundary conditions. Finally, the visualization of streamlines was completed by the ANSYS CFD-Post 14.5 software. The SRV patient had one major right atrioventricular valve and one hypoplastic left atrioventricular valve. The patient had only one ventricular outlet with the aortic valve because of pulmonary atresia (Table 2). As described in other simulations of ventricular flow studies [11–14], in this CFD model, the dynamic valvular motion was not calculated independently with the ventricular walls; similarly, the endocardium was smoothed in the simulated model.

At isovolumic relaxation, the ventricular muscle continues to relax for about 0.03 to 0.06 seconds, and the intraventricular pressures drop rapidly from about 80 mmHg to almost 0 mmHg; then follow the early diastole, with a flow velocity around 1.2 m/s. The blood in the left ventricle is assumed to be an incompressible Newtonian fluid with a constant viscosity of 0.004 kg/ms and density of 1060 kg/m^3^. According to the formula of Reynolds index: Re = *ρ*UD/*μ*, if its Reynolds number > 4000, it is considered to be turbulent. Therefore, the standard *κ*-*ε* model was applied to solve the motion of intraventricular blood flow; this model is a two-equation eddy viscosity model based on the solution of equations for the kinetic energy of turbulence and turbulence dissipation rate. The second-order upwind scheme was employed to complete the steady state numerical simulation by ANSYS-FLUENT 17.0 software. We employed steady state simulation at the three cardiac phases (the end of rapid filling, slow filling, and atrial systole) during diastole to study the cardiac flow status. Finally, boundary conditions from the 2D ECHO data were applied to the finite volume solver package to complete the simulation. When analyzing the vortex during the filling phase, the walls of the models were assumed to be rigid. The blood patterns within the normal RV and SRV were analyzed by the same technique employed for the LV.

During the filling period, the reconstructed and simulated models allowed for the measurements for the dimensions and volume of the ventricles, and the flow dynamics, including maximum length (*L*_LV_, *L*_RV_ or *L*_DVC_), diameter (*D*_*LV*_, *D*_RV_ or *D*_DVC_), and width (*W*_LV_, *W*_RV_ or *W*_DVC_) of the left ventricle (LV), right ventricle (RV), and dominant ventricular chamber (DVC) of the SRV. The ratio of diameter over length of the LV and DVC termed as spherical index (SI) can be derived as well. The area (*A*_MV_, *A*_TV_, or *A*_AV_), perimeter (*P*_MV_, *P*_TV_, or *P*_AV_), and length (*L*_MV_, *L*_TV_, or *L*_AV_) of the mitral valve (MV), tricuspid valve (TV), or atrioventricular valve (AV) and the velocity (*V*_B_, *V*_M_, *V*_A_ or *V*_LB_, *V*_LM_, *V*_LA_, *V*_RB_, *V*_RM_, *V*_RA_) of the base, medium, and apex in the LV, RV, and DVC can be acquired as well. In addition, the volumes of the left ventricle (*V*_LV_), right ventricle (*V*_RV_), and the whole single right ventricle (*V*_SRV_) which was calculated as the summary of the volume of the dominant and rudimentary chambers can be achieved. The CFD models also allow the measurements of end-diastolic volume (EDV), end-systolic volume (ESV), stroke volume (SV), and ejection fraction (EF) of normal LV, RV, and SRV; then the body-surface-area standardized EDV, ESV, SV, and EF were calculated. The flow characteristics inside the SRV were examined and compared with those inside the normal LV and RV. Bland-Altman analysis was performed on diameter and velocity measurements to compare intertechnique agreement between CFD-simulated methods and Echo.

## 3. Results

**Figure 3:**
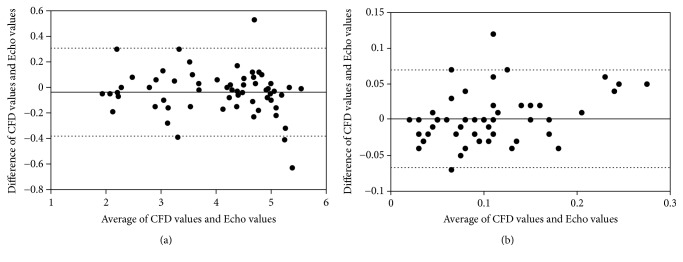
*Bland-Altman analysis of diameter and velocity between CFD and echocardiography measurements*. ((a) Bland-Altman analysis of diameter measurements; (b) Bland-Altman analysis of velocity measurements).

The complexity of the heart anatomy and geometric assumptions of CFD models requires validation of CFD-simulated results, especially in complicated clinical cases. The CFD-simulated results and Echo measured values of the normal subject and the patient were summarized in Tables 3 and 4. The lengths, depths, and widths of the LV, RV, and SRV; SI of the LV and DVC; the areas, perimeters, and lengths of the atrioventricular valve; and the incoming velocities were comparable between the CFD-simulated results and Echo measurements (Figure 3). The qualitative comparisons demonstrated good concordance between the CFD-simulated results and Echo measured values; interclass correlations for diameter and velocity measurements were 0.99 (*p* < 0.01) and 0.84 (*p* < 0.01), respectively.

The volumes measured at different phases of the cardiac cycle in normal LV were equivalent to those in the normal RV (Table 5). Similarly, the BSA calibrated volumes and EF were interchangeable between the normal LV and RV (Table 6). The volumes of the SRV were larger than those of normal LV and RV and much larger when heart failure occurred. The volumes measured at different phases of the cardiac cycle in the SRV demonstrated significant increase when heart failure was present (Table 5). In addition, the BSA calibrated volumes and EF illustrated the impaired functions in SRV when heart failure was present (Table 6). The EF of the SRV without heart failure was comparable to that of normal LV and RV but significantly decreased while suffering heart failure. Spherical index (SI) is the ratio between the short and long axis length, and spherical deformation is the transformation of the ventricle into a spherical shape with an increased circumference relative to the longitudinal deformation. Due to triangular/crescent shape of the normal RV, SI of the RV was not applicable and was not calculated. SI of the LV and the SRV increased during diastole, reached their maximum during atrial systole, and then decreased and reached their minimum during the period of slow ejection. SI of the normal LV during atrial systole is 0.61, whereas those of the SRV, with and without heart failure, were 0.89 and 0.96, respectively, much greater than normal values (Tables 3 and 4).

**Figure 4:**
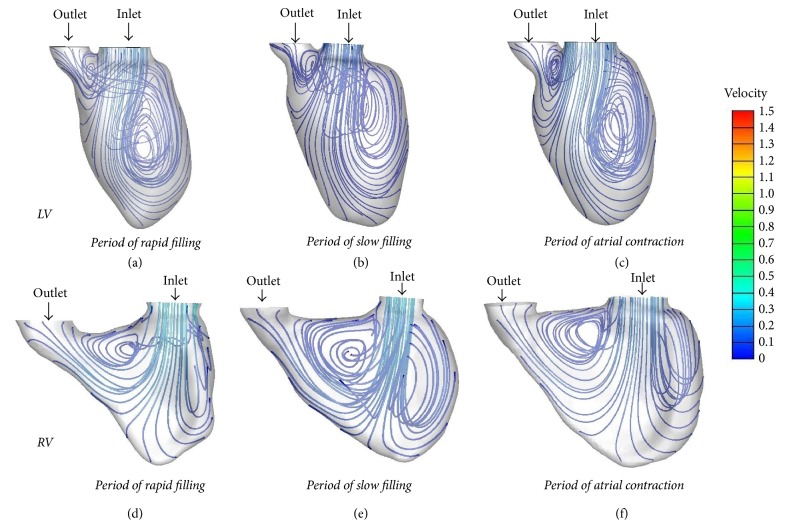
*Streamline in the normal LV and RV during diastole* ((a–c) streamlines in the LV during the rapid filling, slow filling, and atrial systole period; (d–f) streamlines in the RV during the rapid filling, slow filling, and atrial systole period).

**Figure 5:**
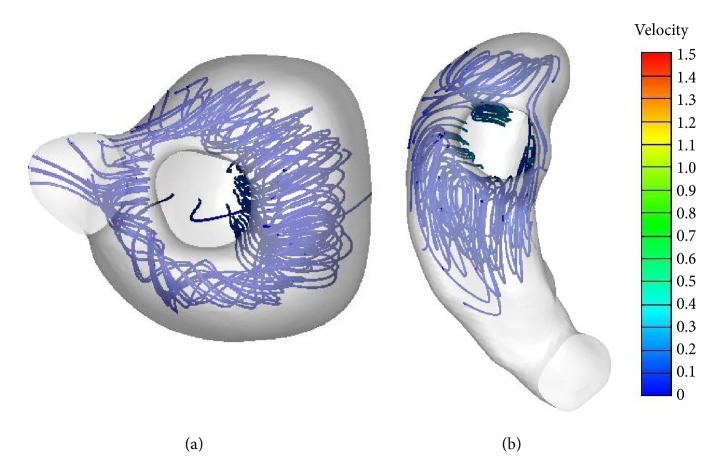
*Vortex rings in the normal LV and RV during diastole* ((a) vertical view of the LV; (b) vertical view of the RV).

CFD enables visualization of the dynamic flow by demonstrating the streamline in the heart, in which the direction and magnitude (color) of the flow can be determined. In normal left ventricle, during the period of rapid filling, blood flew through the mitral valve reaching the apex and swirling, forming a major vortex in the middle of the left ventricle (Figure 4). The major vortex exhibited counterclockwise rotation and persisted throughout the entire diastolic period. During the atrial systolic phase, the vortex was enlarged, occupying almost the entire ventricle. A small clockwise rotation vortex was present at the outflow tract region. A vortex ring was observed in the base region downstream of the inlet orifice (Figure 5).

In normal right ventricle during diastole, the blood went helical and flowed from the tricuspid region toward the free wall and the septal region. The vortex was clockwise and presented at the junction of the tricuspid valve and pulmonary valve; it was larger in size than the vortex inside the LV (Figure 4). Rotating flow was observed near the tricuspid valve, elongating and forming a vortex ring toward the outlet orifice. The RV vortex ring had similar orientation as the LV vortex ring, but with different circularity; it was not as annular as the corresponding LV vortex from vertical view (Figure 5).

**Figure 6:**
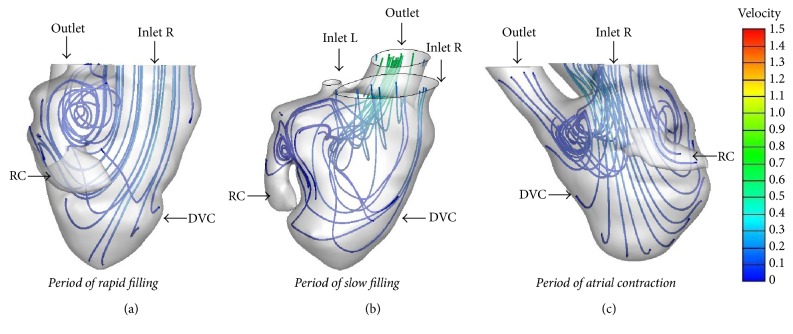
*Streamline in the SRV without heart failure during diastole* ((a) period of rapid filling; (b) period of slow filling; (c) period of atrial systole). DVC: dominant ventricular chamber; RC: rudimentary chamber.

In the SRV without heart failure, during the period of rapid filling, blood flew through the atrioventricular valve and entered the dominant chamber; part of the flow streamed straight ahead to the apex, and part of the flow moved toward the posterior wall to form a vortex near the bulboventricular foramen. The vortex persisted until the slow filling phase and moved toward the interior of the rudimentary chamber. During the atrial systolic phase, a vortex appeared at the aortic root. The vortices in the SRV without heart failure were smaller than those in the normal LV and RV (Figure 6), and no vortex ring was observed in the SRV during diastole.

**Figure 7:**
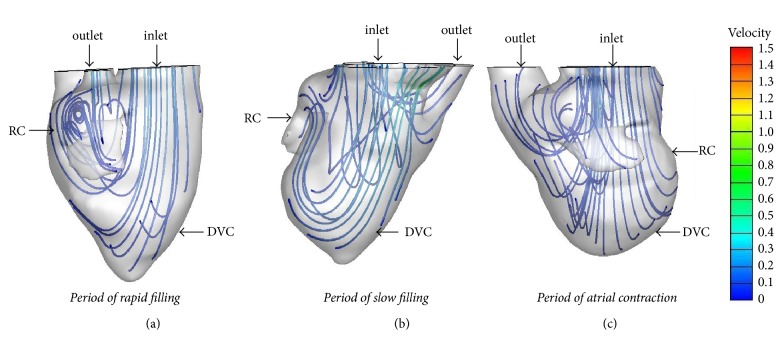
*Streamline in the SRV with heart failure during diastole*. ((a) period of rapid filling; (b) period of slow filling; (c) period of atrial systole). DVC: dominant ventricular chamber; RC: rudimentary chamber.

The vortices changed in the SRV when heart failure was present (Figure 7): there was only one small vortex near the bulboventricular foramen during the rapid filling phase, with rambling streamlines during other diastolic phases. The vortex was smaller than that in SRV without heart failure and normal LV and RV; furthermore, the vortex disappeared during the slow filling and atrial systole period. No vortex ring was observed during diastole in SRV with or without heart failure.

**Figure 8:**
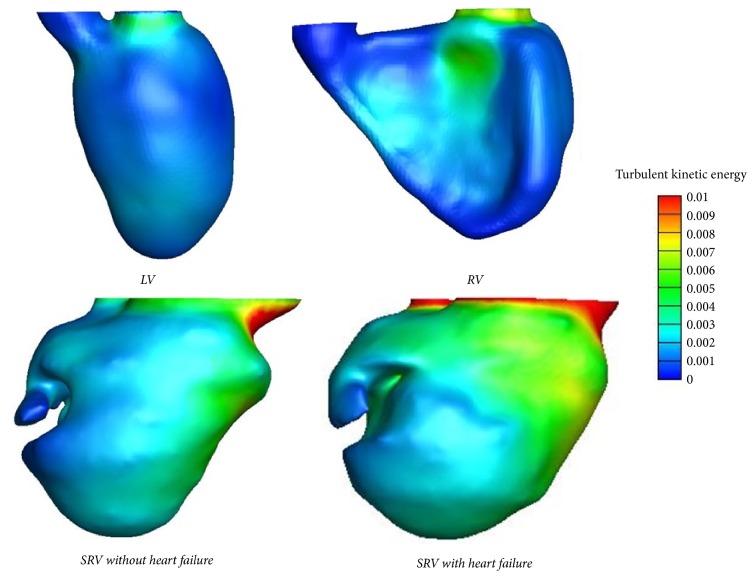
*Turbulent Kinetic Energy (TKE) of LV, RV, and SRV during period of atrial systole*. In normal LV and RV, the TKE values were close to zero; it is concentric and evenly distributed in the ventricle; however, in the SRV, the TKE showed a larger gradient dispersion throughout the ventricle.

Turbulent kinetic energy (TKE) was analyzed in the LV and RV and in the SRV with/without heart failure (Figure 8). In normal LV and RV, the TKE values were close to zero and evenly distributed in the ventricle, while, in the SRV, the TKE showed a larger gradient dispersion throughout the ventricle.

## 4. Discussion

This study applied CFD derived 3D numerical simulations to measure the dimensions, volumes, and functions of ventricles and to visualize the flow dynamics inside of normal LV, RV, and SRV. The qualitative comparisons of dimensions, volumes, and functions showed good agreements between the CFD-simulated results and Echo measured values (Figure 3), which manifested and validated the feasibility and reliability of CFD in monitoring blood flow, geometry, and function of single right ventricle.

Ventricular dysfunction is an important risk factor for the morbidity and mortality in patients with a single ventricle [15]. Blood flow pattern inside the ventricular chamber is related to the functional and morphologic changes of the heart [16]; flow pattern even changes before overt changes occurred in cardiac function.

Previous studies have confirmed that the natural vortex motion in the healthy LV may be corrupted when dysfunctions occur [17]. Liang et al. reported the development of a spherical LV shape accompanying heart failure leading to pronounced deterioration of the pump function of the LV [18]. Therefore, analyzing blood flow dynamics has incremental values in early detection of ventricular dysfunction. The formation of transmitral vortices may be a sensitive marker of diastolic dysfunction [19]. Previous studies [17, 20–22] demonstrated blood flow swirling and forming vortices during the rapid filling phase, which were introduced by both the geometry of the left ventricle and the curvature between the mitral valve and the aorta. Blood flowing through the mitral valve that reaches the apex of the heart would form a major vortex in the middle of the left ventricle. This vortex exhibits counterclockwise rotation and persists throughout diastole. Another clockwise rotation vortex is present near the outflow tract; the formation of this vortex may be due to the expansion of a large cross-sectional area at the junction of the inlet and outlet, which separates the flow. A vortex ring forms near the mitral orifice region, redirecting the flow and preserving the energy. Ventricular relaxation during diastole allows the myocardium to recoil and pump blood into the ventricle. The size and shape of the ventricle may affect the turning of streamlines and the formation of the vortices.

Similar to previous reports [17, 23], we observed that the early inflow goes toward the free wall and the septal region in the normal right ventricle during diastole. A larger clockwise vortex was observed at the junction of the tricuspid valve and pulmonary valve. The formation of this vortex may be due to the funnel shape of RV and the expansion of a large cross-sectional area at the junction of the inlet and outlet which separates the flow. A vortex ring presented near the tricuspid region and elongated to form the helical spinning flow toward the pulmonary orifice. The RV vortex ring was less annular when compared with the LV vortex ring due to the special triangular shape of the RV. Presence of vortex ring may be related to the low pressure gradient inside the right ventricle during diastole. The funnel-like RV geometry assists the flow for ejection by converging toward the pulmonary orifice [5]. Diastolic flow in normal RV makes it more favorable for effective systolic ejection [24].

Myocardial relaxation and contraction are the dynamic energy sources of intracardiac flow. Vortices offer the most appropriate spatial pressure distribution to push the flow. It can impound a certain amount of flow energy during diastolic phase, converting vortex kinetic energy into the rotational kinetic energy by means of gyratory motion, which averts a strong convective deceleration. The diastolic vortices can redirect the flow to avoid excessive dissipation of energy, thereby facilitating ventricular filling and stroke volume maintenance [25]. The natural asymmetric geometry of heart could minimize dissipative interaction of flow convection to arrange the flow for ejection, while unnatural asymmetry flow could reduce the efficiency of the heart pump by more than 10% [26]. Normal flow pattern inside the ventricle is altered in dilated and hypocontractile hearts, accompanied with weakened pumping efficiency [26]. The sphericity of the ventricle makes the atrioventricular annulus more centered or highly eccentric, which may increase dissipation, as reported by Pedrizzetti and Domenichini [26]. It was observed in our study that the turbulent kinetic energy in the SRV was dispersed in the ventricle; however it is more centered and evenly distributed in the normal LV and RV. The abnormal geometry of SRV contributed to the increased dissipation and energy loss of blood flow.

According to previous studies, SRV have reduced systolic and diastolic function compared with those of the respective ventricles in normal biventricular hearts [1]. The SRV has a dominant RV ventricle and a second, hypoplastic rudimentary LV chamber. The main ventricular chamber has a RV anatomy but enlarged to a spherical shape. The morphology of the SRV may be less suitable for the systemic circulation than left ventricle. The vortex during diastole in patient with SRV was much smaller than those in normal LV and RV. When heart failure is present, the vortex inside the ventricle may diminish or even disappear. The myocardial adaptation to chronic systemic pressure has been demonstrated via the transformation of the ventricle into a spherical shape for more efficient contraction [27]. Increased end-systolic chamber size induces atrioventricular valve disproportion with weakening of the vortex. The diminished or obliterated diastolic vortex may increase ventricular work because of decreased energy storage due to the morphologic abnormality of the right ventricle. When natural arrangement is broken, energy dissipation increases, possibly ejecting less energetic blood into the ventricular outlet tract. This reaction may have the consequence of increasing mechanical work of the cardiac muscle to maintain equal energy and eject sufficient amount of flow into the systemic circulation. As a result, it cannot afford the work load; therefore its ventricular myocardium decompensation would result in extra energy dissipation, and ultimately heart dysfunction. Kaneko et al. suggested that the single ventricle with dominant right ventricular morphology may be associated with impaired systolic and diastolic functional parameters [28]; SRV has impaired ventricular function and abnormal flow structure. Smaller or obliterated vortices may prompt the deterioration of ventricular function. CFD may provide a new way of unmasking the myocardium dysfunction in SRV by detecting early changes in the number and shape of the vortices in the ventricle.

To our knowledge, this is the first report of using CFD to evaluate the flow pattern in SRV, but there are some limitations of this study. (1) The CFD analysis in this study was based on echocardiographic images; it suffers the same spatial and temporal resolutions limitations of echocardiography. (2) The SRV papillary muscles and valves were smoothing out to optimize CFD modeling. In spite of its limitations, CFD still can provide detailed real-time information about complex intraventricular flow pattern, leading to a better understanding of both physiological and pathological hemodynamics in SRV.

## 5. Conclusion

In summary, CFD 3D modeling can not only evaluate the morphological and functional abnormalities in SRV, but also detect the abnormal flow pattern and vortices during diastole. CFD shows promising role as an additional noninvasive tool to assist conventional clinical techniques in visualizing flow in the heart as well as gain further understanding and optimize their treatment.

## Figures and Tables

**Table 1:** Basic demographic information and clinical characteristics of the study subjects.

	Control	SRV	SRV + HF
Age (y)	4	4	4
Gender	Male	Male	Male
Height (cm)	100	101	107
Weight (Kg)	15.0	15.0	19.0
BSA (m^2^)	0.65	0.65	0.75
HR (beat/min)	118	92	90
BP (mmHg)	98/56	108/58	100/63

*Note*. BSA: body surface area; HR: heart rate; BP: blood pressure; SRV: single right ventricle without heart failure; SRV + HF: single right ventricle with heart failure.

**Table 2:** Boundary conditions of the CFD-simulation models (inlet and outlet velocities).

Velocity (m/s)	PRF	PSF	PAS	PRE	PSE
Control					
Inlet L	0.21	0.14	0.20	0.00	0.00
Outlet L	0.00	0.00	0.00	1.00	0.35
Inlet R	0.20	0.20	0.18	0.00	0.00
Outlet R	0.00	0.00	0.00	0.90	0.20
SRV					
Inlet L	0.05	0.10	0.05	0.00	0.00
Inlet R	0.17	0.18	0.12	0.00	0.00
Outlet	0.00	0.00	0.00	0.89	0.38
SRV + HF					
Inlet L	0.25	0.20	0.15	0.00	0.00
Inlet R	0.20	0.20	0.12	0.00	0.00
Outlet	0.00	0.00	0.00	0.90	0.25

*Note*. CFD: computational flow dynamics; SRV: single right ventricle without heart failure; SRV + HF: single right ventricle with heart failure; PFR: period of rapid filling; PSF: period of slow filing; PAS: period of atrial systole; PRF: period of rapid ejection; PSE: period of slow ejection; Inlet L: left inlet, mitral valve or left atrioventricular valve level; Inlet R: right inlet, tricuspid valve or right atrioventricular valve level; Outlet L: left outlet, aortic valve level; Outlet R: right outlet, pulmonary valve level.

**Table 3:** Percentage differences between CFD-simulated values and ECHO measured values in the normal LV and RV.

LV	Percentage differences					RV	Percentage differences				
	PRF	PSF	PAS	PRE	PSE		PRF	PSF	PAS	PRE	PSE
*L*_LV_ (cm)	1.72	−4.23	0	0.47	1.5	*L*_RV_ (cm)	2.09	0.60	−0.18	−1.00	−1.35
*D*_LV_ (cm)	−5.05	−4.98	1.55	3.28	−1.79	*D*_RV_ (cm)	−8.56	−3.10	0.00	−2.38	−2.55
*W*_LV_ (cm)	0	4.38	9.43	2.08	14.63	*W*_RV_ (cm)	1.57	−3.70	−0.41	0.45	−0.68
SI	−6.25	−1.61	1.67	3.51	1.79	*A*_TV_ (cm^2^)	0.26	−0.48	3.42	1.97	−13.62
*A*_MV_ (cm^2^)	2.01	−2.35	−1.16	0.45	−1.47	*P*_TV_ (cm)	0.14	−0.13	1.58	−0.26	−0.14
*P*_MV_ (cm)	−0.61	−2.83	−2.38	0.62	1.21	*L*_TV_ (cm)	−2.11	−2.07	3.52	0.41	3.56
*L*_MV_ (cm)	2.34	−3.26	−5.84	3.60	3.91	*V*_B_ (m/s)	18.18	−11.11	−25.00	/	/
*V*_B_ (m/s)	13.33	0	15.38	/	/	*V*_M_ (m/s)	30.00	−26.67	−40.00	/	/
*V*_M_ (m/s)	0	0	0	/	/	*V*_A_ (m/s)	240.00	233.33	0.00	/	/
*V*_A_ (m/s)	−50	−60	0	/	/						

*Note*. Positive values: CFD-simulated values greater than the ECHO measured values; negative values: CFD-simulated values smaller than the ECHO measured values; CFD: computational flow dynamics; ECHO: echocardiography; LV: left ventricle; PFR: period of rapid filling; PSF: period of slow filing; PAS: period of atrial systole; PRF: period of rapid ejection; PSE: period of slow ejection; *L*_LV_: length of the left ventricle; *D*_LV_: diameter of the left ventricle; *W*_LV_: width of the left ventricle; SI: spherical index; *A*_MV_: area of the mitral valve; *P*_MV_: perimeter of the mitral valve; *L*_MV_: length of the mitral valve; *V*_B_: basal velocity in the left ventricle; *V*_M_: medial velocity in the left ventricle; *V*_A_: apical velocity in the left ventricle. RV: right ventricle; *L*_RV_: length of the right ventricle; *D*_RV_: diameter of the right ventricle; *W*_RV_: width of the right ventricle; *A*_TV_: area of the tricuspid valve; *P*_TV_: perimeter of the tricuspid valve; *L*_TV_: length of the tricuspid valve.

**Table 4:** Percentage differences between CFD-simulated values compared with ECHO measured values in the SRV with and without heart failure (HF).

No HF	Simulated values					HF	ECHO measured values				
	PRF	PSF	PAS	PRE	PSE		PRF	PSF	PAS	PRE	PSE
*L*_DVC_ (cm)	−0.20	−0.59	−1.15	11.96	3.95		−1.98	−7.52	−11.05	2.54	0.64
*D*_DVC_ (cm)	−4.04	−1.87	−4.79	−0.54	−8.59		0.00	−0.47	−1.61	0.82	2.84
*W*_DVC_ (cm)	−3.37	−2.33	−3.09	5.85	−3.23		−0.89	2.61	−5.90	−4.16	−11.14
SI	−3.53	−1.19	−3.26	−11.90	−11.84		2.41	7.59	10.34	−2.56	2.67
*A*_AV_ (cm^2^)	−6.46	−7.43	0.39	5.94	16.13		2.91	1.91	−1.66	−0.91	3.69
*P*_AV_ (cm)	−10.99	−7.42	−5.22	−1.19	−0.89		−3.53	−2.57	−1.94	−0.15	2.15
*L*_AV_ (cm)	−1.00	0.75	−8.45	−1.49	−1.11		−4.24	−1.85	−6.11	−2.64	0.25
*V*_LB_ (m/s)	−60.00	20.00	−20.00	/	/		9.09	0.00	−80.00	/	/
*V*_LM_ (m/s)	−12.50	0.00	0.00	/	/		−70.00	−25.00	−40.00	/	/
*V*_LA_ (m/s)	0.00	60.00	0.00	/	/		−50.00	75.00	25.00	/	/
*V*_RB_ (m/s)	0.00	0.00	14.29	/	/		−20.00	22.73	−9.09	/	/
*V*_RM_ (m/s)	−16.67	0.00	77.78	/	/		−20.00	20.00	−27.27	/	/
*V*_RA_ (m/s)	0.00	−27.27	66.67	/	/		−20.00	5.00	−12.50	/	/

*Note*. Positive values: CFD-simulated values greater than the ECHO measured values; negative values: CFD-simulated values smaller than the ECHO measured values; CFD: computational flow dynamics; ECHO: echocardiography; SRV: single right ventricle; PFR: period of rapid filling; PSF: period of slow filing; PAS: period of atrial systole; PRF: period of rapid ejection; PSE: period of slow ejection; *L*_DVC_: length of the dominate ventricular chamber; *D*_DVC_: diameter of the dominate ventricular chamber; *W*_DVC_: width of the dominate ventricular chamber; SI: spherical index; *A*_AV_: area of the atrioventricular valve; *P*_AV_: perimeter of the atrioventricular valve; *L*_AV_: length of the atrioventricular valve; *V*_LB_: basal velocity of the left atrioventricular valve; *V*_LM_: medial velocity of the left atrioventricular valve; *V*_LA_: apical velocity of the left atrioventricular valve; *V*_RB_: basal velocity of the right atrioventricular valve; *V*_RM_: medial velocity of the right atrioventricular valve; *V*_RA_: apical velocity of the right atrioventricular valve.

**Table 5:** CFD derived volume measurements of the LV, RV, and SRV.

Volume (ml)	PRF	PSF	PAS	PRE	PSE
Control					
*V*_LV_	24.20	27.10	39.01	23.81	15.50
*V*_RV_	28.11	29.37	40.25	26.41	16.74
SRV					
*V*_SV_	48.35	50.53	63.86	37.98	27.56
SRV + HF					
*V*_SV_	51.96	56.06	68.64	49.54	42.38

*Note*. CFD: computational flow dynamics; LV: left ventricle; RV: right ventricle; SRV: single right ventricle without heart failure; SRV + HF: single right ventricle with heart failure; PFR: period of rapid filling; PSF: period of slow filing; PAS: period of atrial systole; PRF: period of rapid ejection; PSE: period of slow ejection; *V*_LV_: volume of the left ventricle; *V*_RV_: volume of the right ventricle; *V*_SV_: volume of the whole functional single ventricle.

**Table 6:** BSA calibrated volume and EF of the normal child and the patient.

	EDV (ml/m^2^)	ESV (ml/m^2^)	SV (ml/m^2^)	EF (%)
Control (LV)	60.01	23.85	36.16	60.26
Control (RV)	61.92	25.75	36.17	58.41
SRV	98.25	42.40	55.85	56.84
SRV + HF	91.52	56.51	35.01	38.25

*Note*. BSA: body surface area; SRV: single right ventricle without heart failure; SRV + HF: single right ventricle with heart failure; LV: left ventricle; RV: right ventricle; EDV: end-diastolic volume; ESV: end-systolic volume; SV: stroke volume; EF: ejection fraction.
